# Supplementary material for: Formation of organic color centers in air-suspended carbon nanotubes using vapor-phase reaction
Source: Nat Commun. 2022 May 20;13:2814. doi: 10.1038/s41467-022-30508-z (PMC9123200; doi:10.1038/s41467-022-30508-z)
Supplement: Supplementary file 1 — Supplementary Information [file 41467_2022_30508_MOESM1_ESM.pdf]

Supplementary Information: Formation of Organic Color Centers in Air-Suspended Carbon Nanotubes Using Vapor-Phase Reaction

D. Kozawa et al.

Supplementary Figures

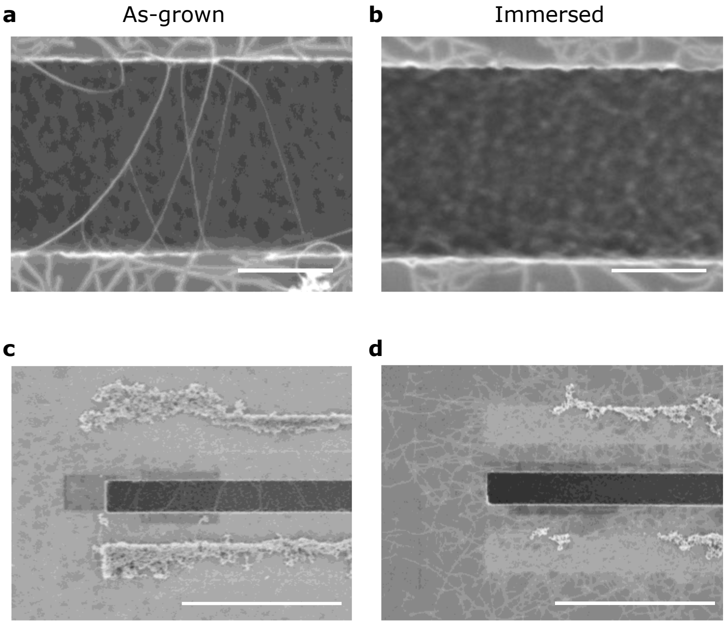

Supplementary Figure 1 | Effect of water immersion. Scanning electron micrographs of suspended SWCNTs across a trench with ta width of 1.0 μm, which are taken in representative regions for (a) before and (b) after immersing the sample in water for 60 s and dried on a hot plate at 80 °C for 60 s. Panels (c) and (d) are zoomed-out images of trenches (c) before and (d) after the immersion, respectively. The scale bars in (a,b) and (c,d) are 500 nm and 5 μm, respectively. Source data are provided as a Source Data file.

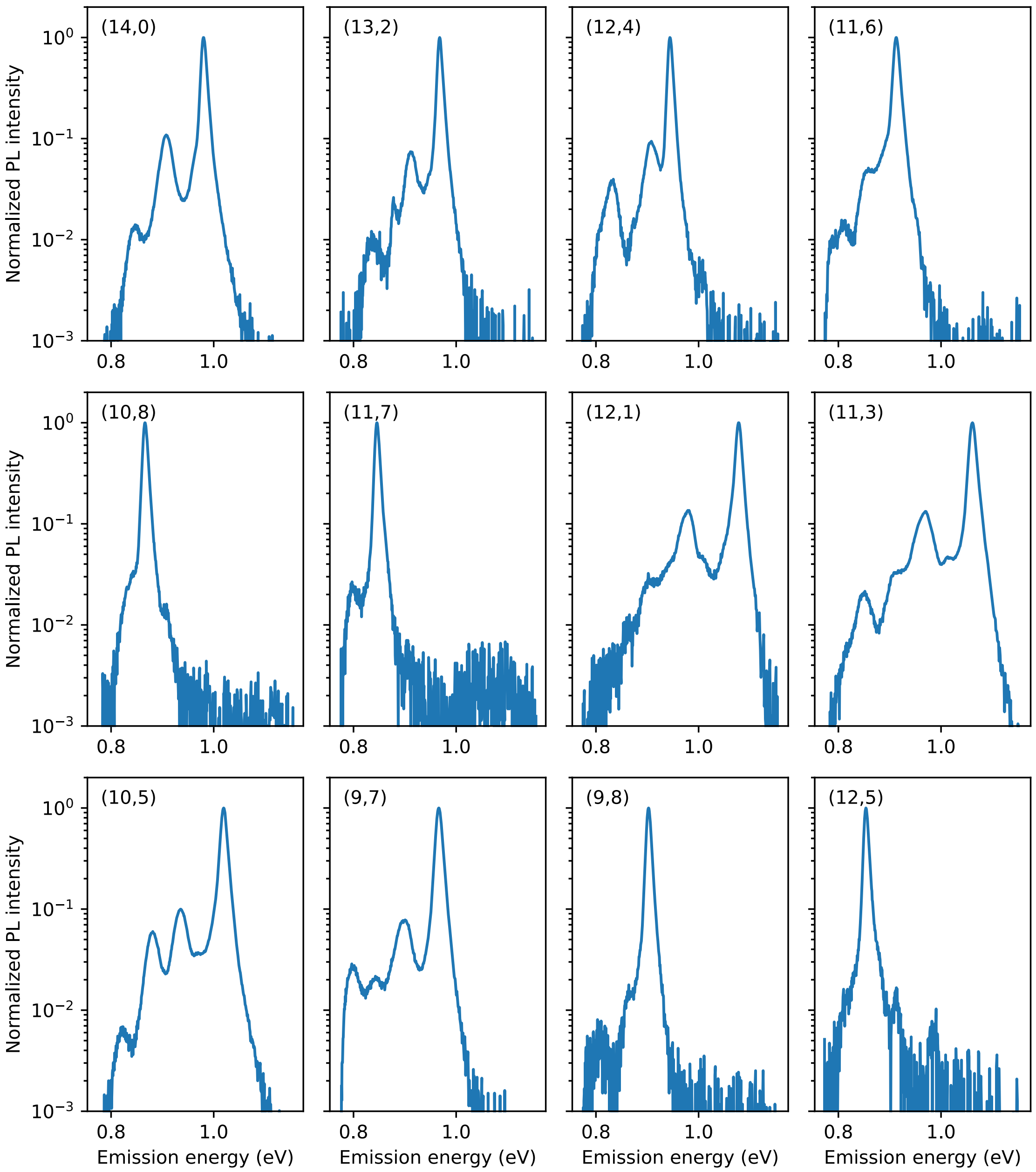

Supplementary Figure 2 | PL spectra in a logarithmic scale. Representative PL spectra of functionalized SWCNTs measured with the same excitation condition as Fig. 2, where the vertical axis is in a logarithmic scale. Smaller peaks at energies lower than E_{11}^{-*} may be caused by color centers with a different binding configuration. Source data are provided as a Source Data file.

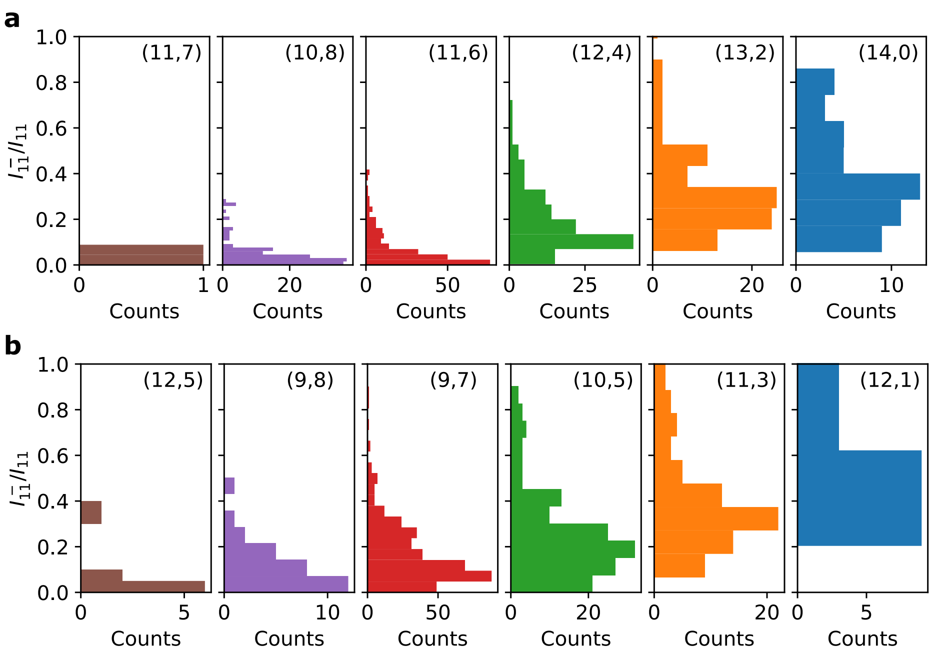

Supplementary Figure 3 | Distribution of subpeak ratio. Histograms of the subpeak ratio for experiments performed with excitation energies of (a) 1.46 and (b) 1.59 eV and an excitation power of 100 µW. Asymmetric distributions with long upper tails are observed for all the chiralities, indicating that relatively unreacted tubes are more abundant. The bin widths of this histogram and all the following are chosen by Freedman Diaconis Estimator which considers data variability and size. Source data are provided as a Source Data file.

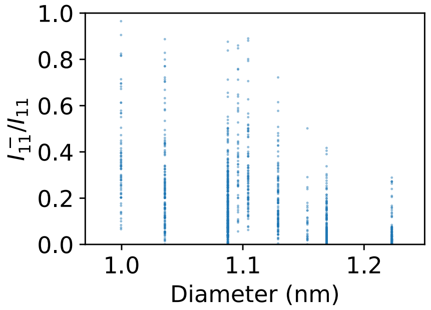

Supplementary Figure 4 | Variation of subpeak ratios. Diameter dependence of subpeak ratio for experiments conducted with excitation energies of 1.46 and 1.59 eV and an excitation power of 100 µW. Source data are provided as a Source Data file.

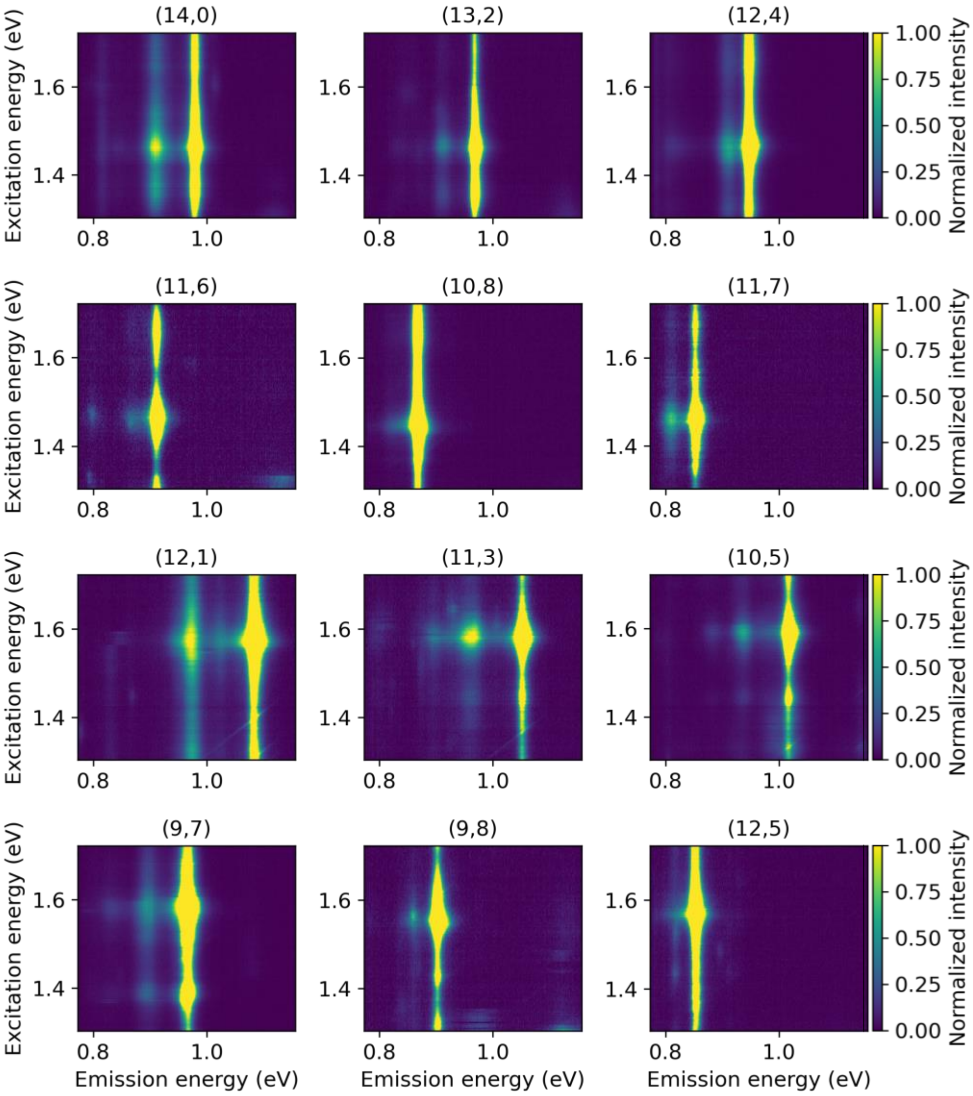

Supplementary Figure 5 | Resonance energy. PL excitation maps for various chiralities of functionalized SWCNTs measured with an excitation power of 100 μW. Source data are provided as a Source Data file.

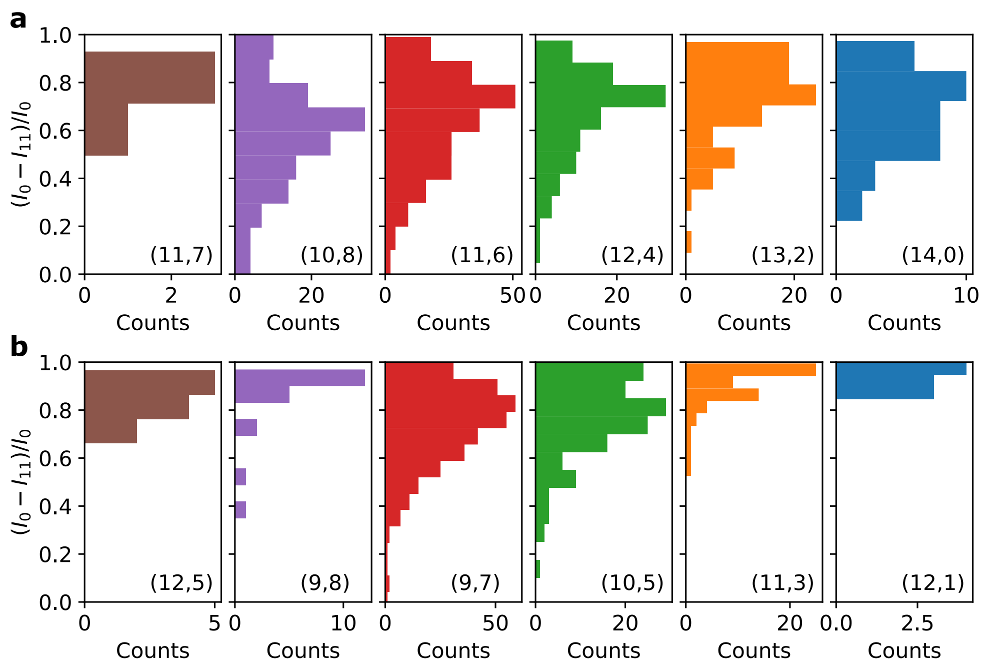

Supplementary Figure 6 | Distribution of quenching degree. Histograms of the quenching degree for experiments conducted with excitation energies of (a) 1.46 and (b) 1.59 eV and an excitation power of 10 µW. Source data are provided as a Source Data file.

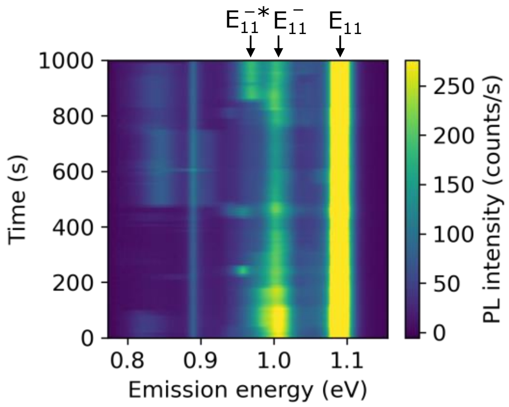

Supplementary Figure 7 | PL intermittency. A PL time trace of a functionalized (11,3) SWCNT exposed for 10 s each at the same spot over 1000 s, where the measurements are performed with an excitation energy of 1.59 eV and a power of 100 µW. There are temporal fluctuations in the intensity for E_{11}^{-} and E_{11}^{-*}, but they are intermittent blinking that can be explained by trapping and detrapping of surface charge[1] and do not indicate irreversible reaction. A sharp line at 0.89 eV is likely E_{11} emission from a tube in the vicinity with a different chirality. Source data are provided as a Source Data file.

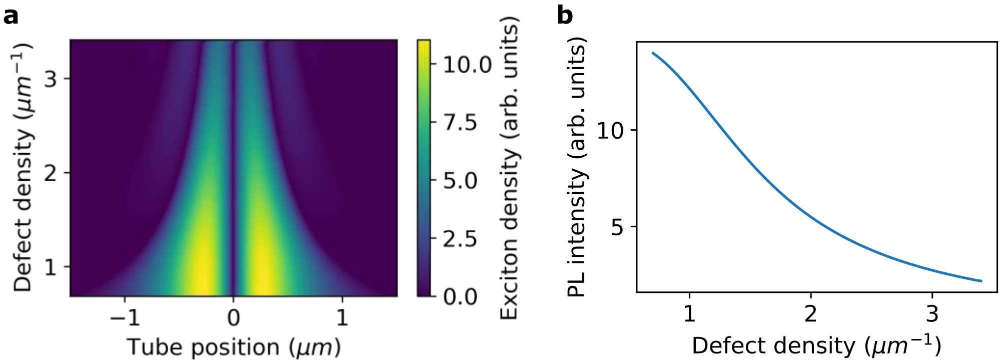

Supplementary Figure 8 | Simulated exciton density and PL intensity. Simulations of defect density dependence on (a) exciton density spatial profile and (b) PL intensity for functionalized tubes with a diameter of 1.00 nm. Source data are provided as a Source Data file.

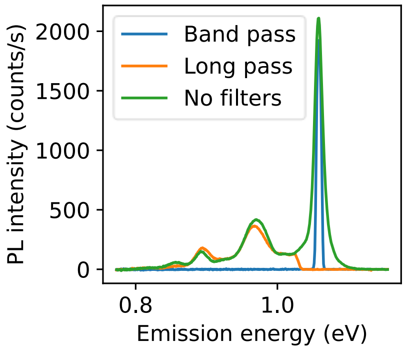

Supplementary Figure 9 | Filtered PL spectra. PL spectra of a functionalized (11,3) SWCNT measured with a band-pass filter (blue) or a long-pass filter (orange) to differentiate E_{11} emission from E_{11}^{-} and E_{11}^{-*} emission. A PL spectrum obtained without the filters is also shown in green. Source data are provided as a Source Data file.

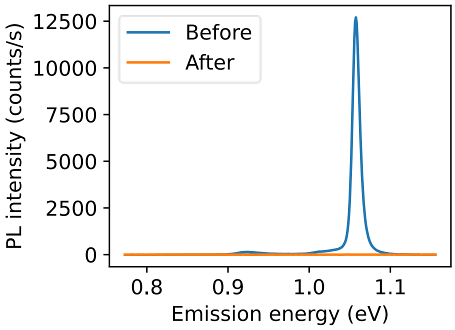

Supplementary Figure 10 | Longer reaction time. Representative PL spectra of an identical air-suspended (11,3) SWCNT before and after the functionalization with longer reaction time measured using a laser power of 10 μW and an excitation energy of 1.59 eV. The spectra show that the longer reaction time results in losing almost the entire fluorescence signal, presumably due to formation of high-density quenching sites. Source data are provided as a Source Data file.

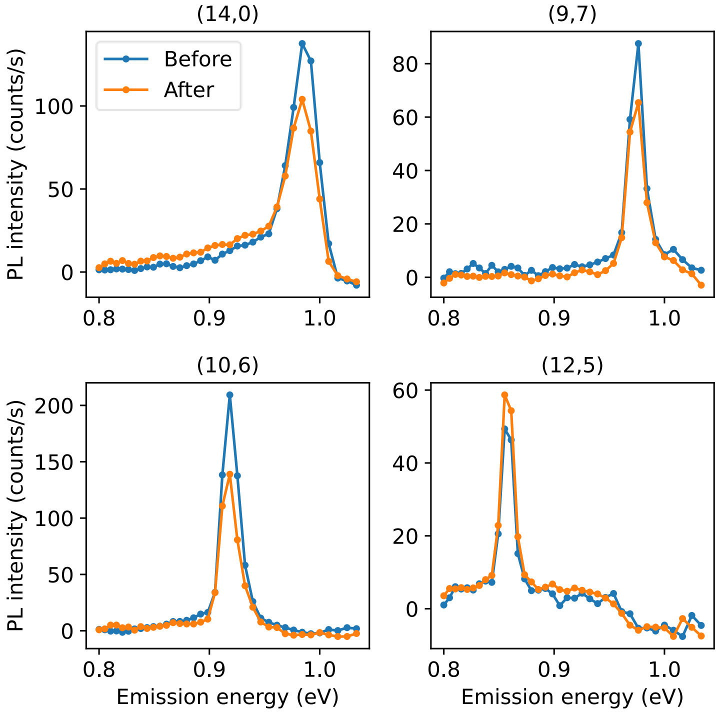

Supplementary Figure 11 | UV irradiation in the absence of iodobenzene. Representative PL spectra for the same nanotubes before and after UV irradiation (4 minutes, photon energy 4.88 eV) in the absence of iodobenzene. The experiments are performed using hyperspectral

imaging with an excitation energy of 1.70 eV with the same configuration as Ref. 1. Chiralities of the tubes are indicated on top of the panels. Source data are provided as a Source Data file.

Supplementary Note 1. Variability in I_0

PL intensity from suspended nanotubes depends strongly on their suspended lengths, and variation of the suspended length causes spread of I_0 (Supplementary Figs. 12, 13 and 14).

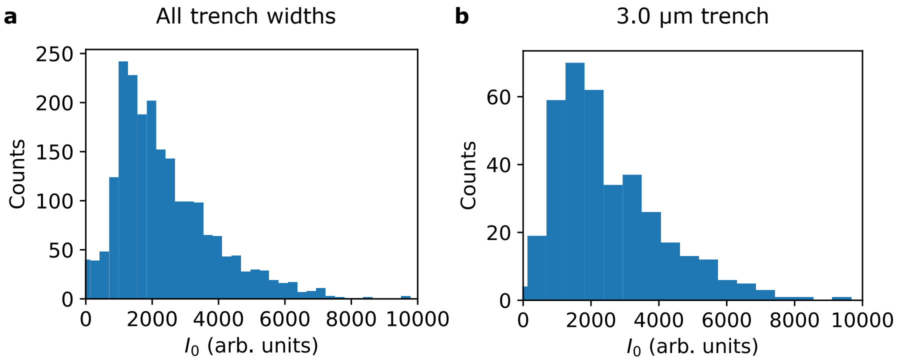

Supplementary Figure 12 | Variability in I_0. Distribution of I_0 for nanotubes with all the chiralities, which are suspended across (a) all the trench widths from 0.5 to 3.0 μm and (b) 3.0 μm. The data are collected with nearly resonant excitation energies of 1.46 or 1.59 eV and a power of 10 μW. Source data are provided as a Source Data file.

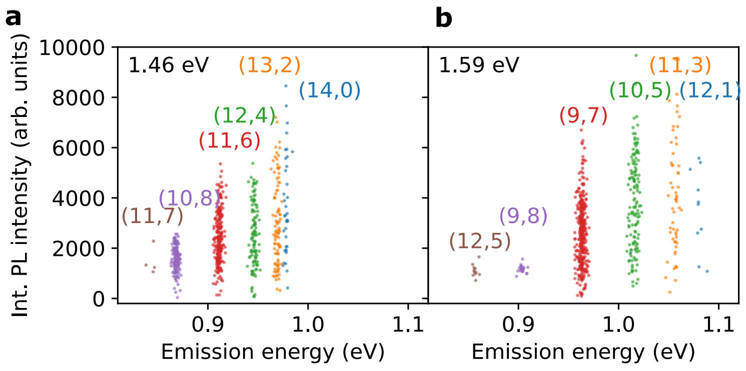

Supplementary Figure 13 | Emission energy dependence of variability in I_0. Spectrally integrated PL intensity I_0 as a function of emission energy for experiments conducted with excitation energies of (a) 1.46 and (b) 1.59 eV and an excitation power of 10 μW. Source data are provided as a Source Data file.

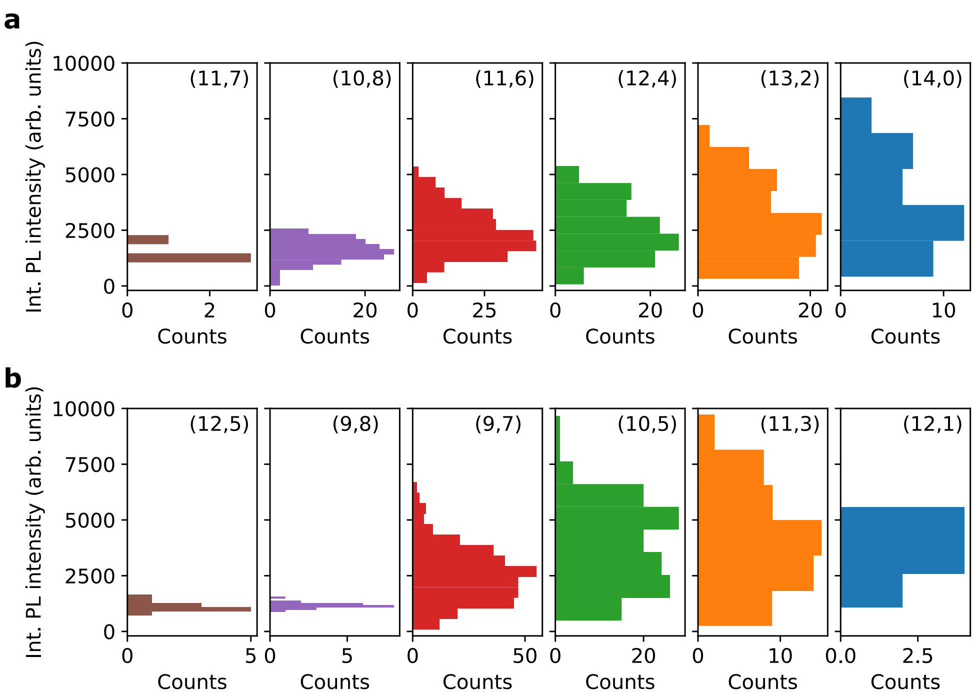

Supplementary Figure 14 | Chirality dependence of variability in I_0. Histograms of spectrally integrated PL intensity I_0 for experiments conducted with excitation energies of (a) 1.46 and (b) 1.59 eV and an excitation power of 10 μW. The bin width is chosen by Freedman Diaconis Estimator which takes into account data variability and size. Source data are provided as a Source Data file.

The suspended length can vary when nanotubes are not fully suspended as seen in PL images (Supplementary Fig. 15).

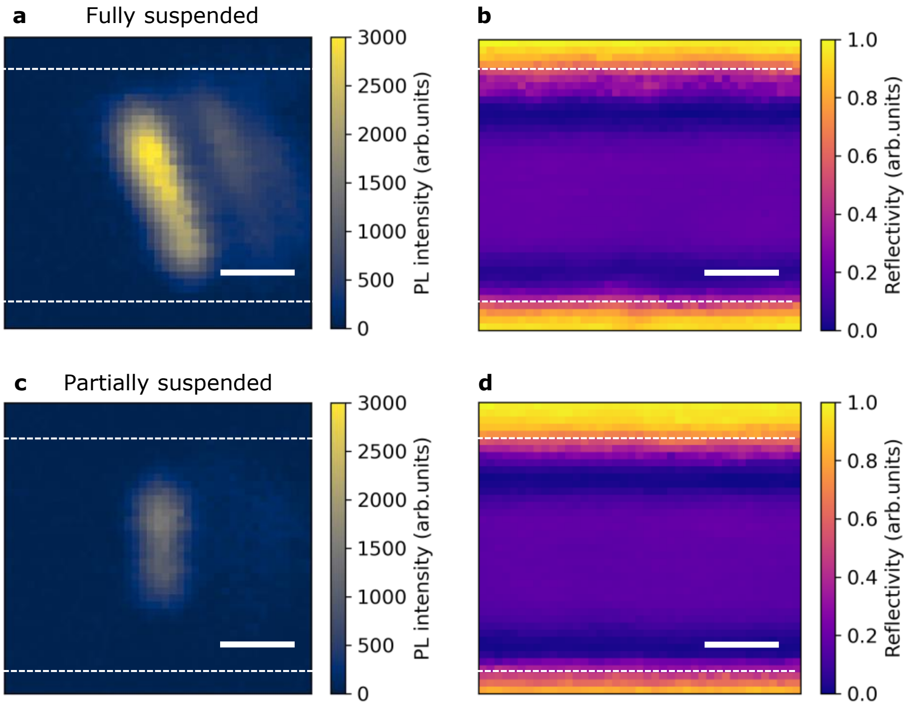

Supplementary Figure 15 | Effect of the suspended length. (a) A representative PL intensity map of E_{11} emission and (b) reflection image in the same area for a fully suspended SWCNT across a trench with a width of 3.0 μm. (c) PL intensity map and (d) reflection image in the same area for a short suspended SWCNT across a trench with the same width where a fallen region is indicated by an arrow. The PL data are collected with an excitation energy of 1.59 eV and a power of 100 μW. The scale bars are 2.0 μm and the white broken lines indicate the edges of the trenches. Source data are provided as a Source Data file.

The length dependent PL intensity can be understood by a one-dimensional exciton diffusion model[2,3]. After generation of excitons upon photoexcitation, excitons diffuse until either radiative recombination or quenching takes place. For small L, most excitons diffuse to unsuspended regions before radiative recombination which ~~and~~ results in quenching. As L gets larger, less excitons reach the ends and more excitons radiatively recombine, resulting in the increased PL intensity. Such length dependence of PL intensity combined with a distribution of L results in the intensity variation.

Simulations of I_0 is performed to interpret the distribution of I_0. We assume log-normal distribution[4] of L

$$\frac{1}{\sigma L\sqrt{2\pi}}\exp\left(-\frac{(\ln(L)-\mu)^2}{2\sigma^2}\right)$$

where σ is the standard deviation and μ is the mean log of the length (Supplementary Fig. 16a).

PL intensity I_0 for each L with defect free initial condition is computed as shown in Supplementary Fig. 16b. When $\mu = -0.25$ corresponding to 0.78 μm, the simulation reproduces the experiments that show an asymmetric peak with a longer tail towards higher I_0.

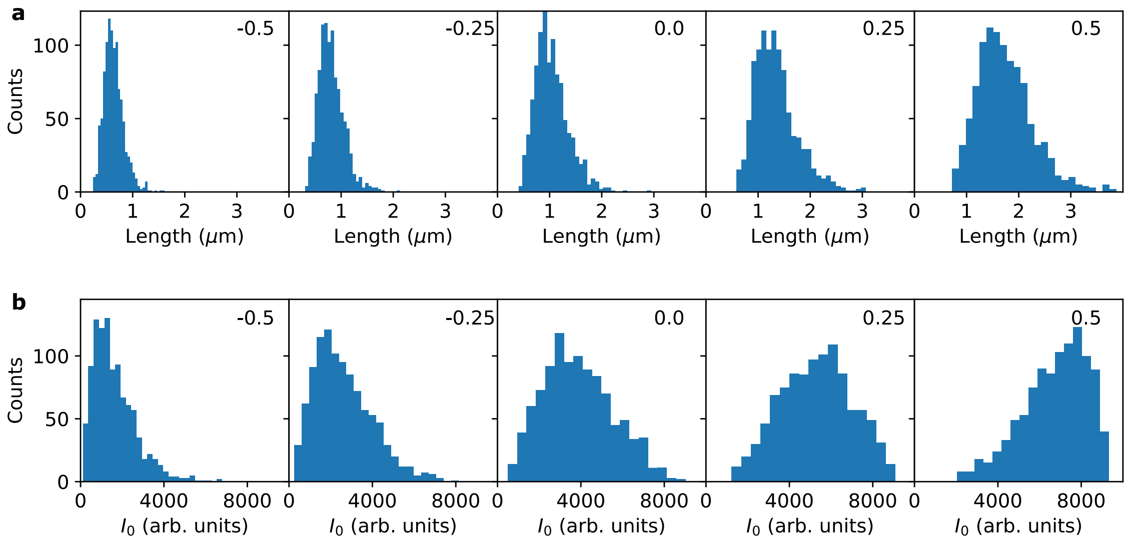

Supplementary Figure 16 | Simulated length and intensity distribution. (a) Randomly generated log-normal distribution of the tube length with $\sigma = 0.3$ and various μ. (b) Simulated PL intensity for each L. The value of μ is indicated on the top right, and I_0 is multiplied by a factor to match the experimental values. Source data are provided as a Source Data file.

To examine if existence of initial defects can cause the spread of I_0, we also conduct simulations of I_0 with various initial defect densities (Supplementary Fig. 17). None of the simulations show characteristic features of an asymmetric peak with a longer tail towards higher I_0, and thus the assumption of the initial defects is inconsistent with the experimental results (Supplementary Fig. 12).

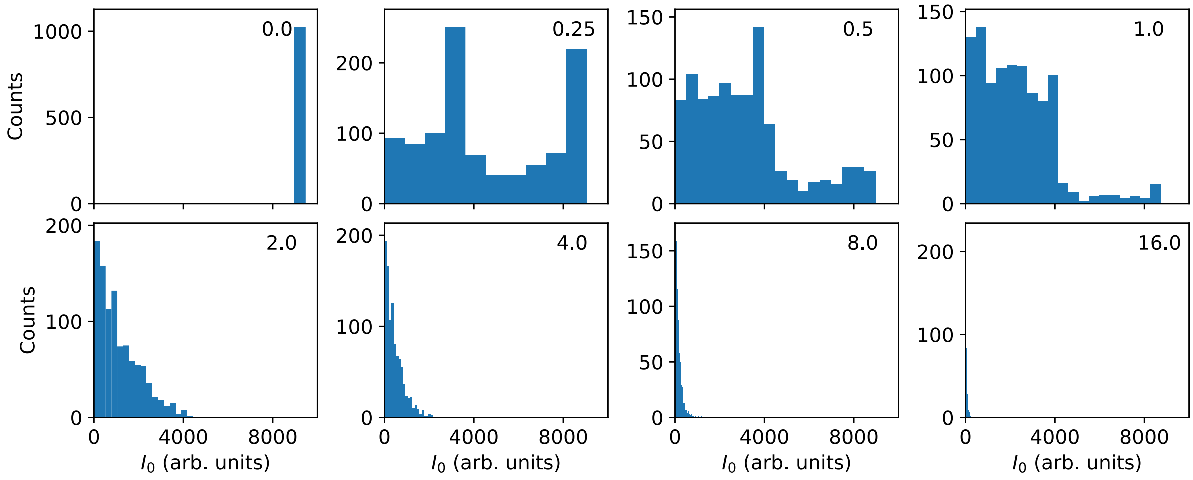

Supplementary Figure 17 | Effect of initial defect densities. Distribution of simulated PL intensities for 3.0-µm-long nanotubes with various initial defect densities from 0.0 to 16.0 µm^{-1}. The initial defect density is indicated on the top right, and I_0 is multiplied by a factor that matches the experimental values. Source data are provided as a Source Data file.

Supplementary Note 2. Excitation power dependence of functionalized tubes

The PL spectra with an excitation power of 100 µW are used to analyze the E_{11}^{-} or E_{11}^{-*} peak parameters because the signal-to-noise at the excitation power of 10 µW is insufficient to accurately extract the peak intensity and position. The stronger saturation for E_{11}^{-} (Supplementary Fig. 18) is attributed to ground-state depletion of E_{11}^{-} exciton states under an ample supply of E_{11} excitons[5], which could lead to underestimation of the reactivity for forming color centers and defect density. We recognize the limitation and thus use the quenching ratio instead of the subpeak ratio to estimate the defect density.

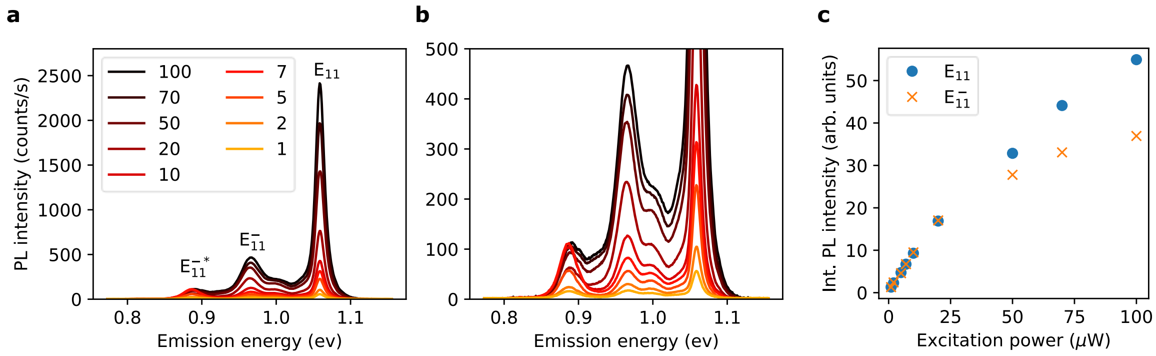

Supplementary Figure 18 | Power dependent PL. (a) Excitation power dependence of PL spectra with an excitation energy of 1.59 eV for functionalized (11,3) tubes. (b) Zoomed-in spectra of (a). Emission peaks in the spectra show negligible broadening and spectral shift. (c) Power dependence of spectrally integrated PL intensity for E_{11} and E_{11}^{-} emission peaks which exhibits linear response up to 20 μW. Source data are provided as a Source Data file.

Supplementary Note 3. Effect of spatial inhomogeneity of functionalization

The spatial inhomogeneity of functionalization as observed in Figs. 1e and 1f may affect the estimation of the defect density. Our model for an exciton density profile in a functionalized tube assumes that a defect is always formed at the center of a nanotube and other defects are created with even separations. This defect distribution results in the smallest PL intensity among all different distributions with the same number of defects, and thus the estimated defect density is a lower bound.

Supplementary Note 4. Distribution of (I_0−I_{11})/I_0

We examine the distribution of the quenching ratio to obtain additional insights regarding the defect density. Experimental results of $(I_0-I_{11})/I_0$ as a function of I_0 show a peak at $(I_0-I_{11})/I_0$ ~0.75 with a large spread (Supplementary Fig. 19). We compare with simulations performed for both low and high defect density regimes. The quenching degree for defect density ρ_{add} is computed using nanotubes with the log-normal distribution of L, where defects are randomly positioned. A simulation result with low ρ_{add} well reproduces the experiments (Supplementary Fig. 20a), resulting in a scatter plot without a consistent trend. In contrast, a simulation result with high ρ_{add} shows saturation of $(I_0-I_{11})/I_0$ at 1.0 (Supplementary Fig. 20b) being inconsistent with the experiments.

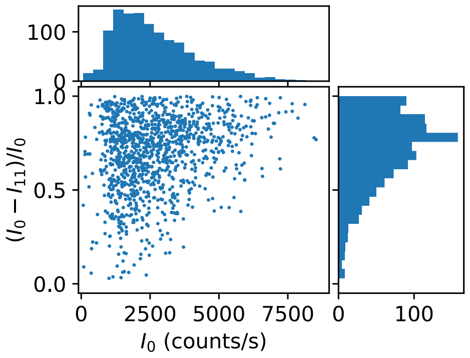

Supplementary Figure 19 | Distribution of the quenching degree (I_0−I_{11})/I_0. Experimentally obtained (I_0−I_{11})/I_0 as a function of I_0 along with histograms for all the 12 chiralities, where the data are collected with nearly resonant excitation energies of 1.46 or 1.59 eV and a power of 10 μW. Source data are provided as a Source Data file.

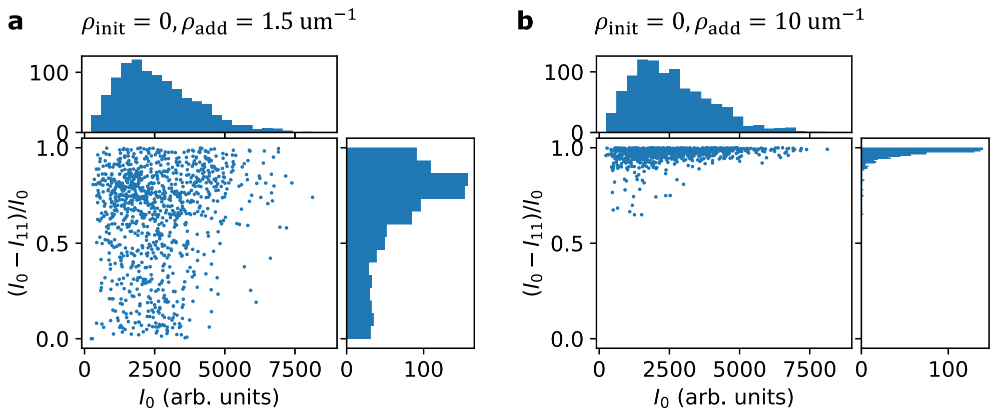

Supplementary Figure 20 | Simulated distribution of (I_0−I_{11})/I_0. Computed quenching degrees as a function of I_0 along with histograms where functionalization with (a) ρ_{add} = 1.5 μm^{-1} and (b) 10 μm^{-1} for defect-free nanotubes with the log-normal distribution of L with μ = −0.25 is simulated. Source data are provided as a Source Data file.

Supplementary Note 5. Similarity to triplet states

The 1/d^2 dependence is also observed in singlet-triplet energy separation $\Delta E_{\mathrm{S-T}}$ extracted from PL and optically detected magnetic resonance spectra[6]. $\Delta E_{\mathrm{S-T}}$ is 30 meV for tubes with a diameter of 1 nm which is smaller than ΔE_{11}^{-} and ΔE_{11}^{-*} for corresponding air-suspended tubes (Figs. 5c and 5d), presumably due to the difference in the dielectric environment.

Supplementary References

1. Wu, X., Kim, M., Qu, H. & Wang, Y. H. Single-defect spectroscopy in the shortwave infrared. *Nat. Commun.* **10**, 1–7 (2019).

2. Ishii, A., Yoshida, M. & Kato, Y. K. Exciton diffusion, end quenching, and exciton-exciton annihilation in individual air-suspended carbon nanotubes. *Phys. Rev. B* **91**, 125427 (2015).

3. Moritsubo, S. *et al.* Exciton diffusion in air-suspended single-walled carbon nanotubes. *Phys. Rev. Lett.* **104**, 1–4 (2010).

4. Wang, S., Liang, Z., Wang, B. & Zhang, C. Statistical characterization of single-wall carbon nanotube length distribution. *Nanotechnology* **17**, 634–639 (2006).

5. Iwamura, M. *et al.* Nonlinear Photoluminescence Spectroscopy of Carbon Nanotubes with Localized Exciton States. *ACS Nano* **8**, 11254–11260 (2014).

6. Palotás, J. *et al.* Incidence of Quantum Confinement on Dark Triplet Excitons in Carbon Nanotubes. *ACS Nano* **14**, 11254–11261 (2020).
