# Supplementary figures and images for: Formation of organic color centers in air-suspended carbon nanotubes using vapor-phase reaction
Source: Nat Commun. 2022 May 20;13:2814. doi: 10.1038/s41467-022-30508-z (PMC9123200; doi:10.1038/s41467-022-30508-z)

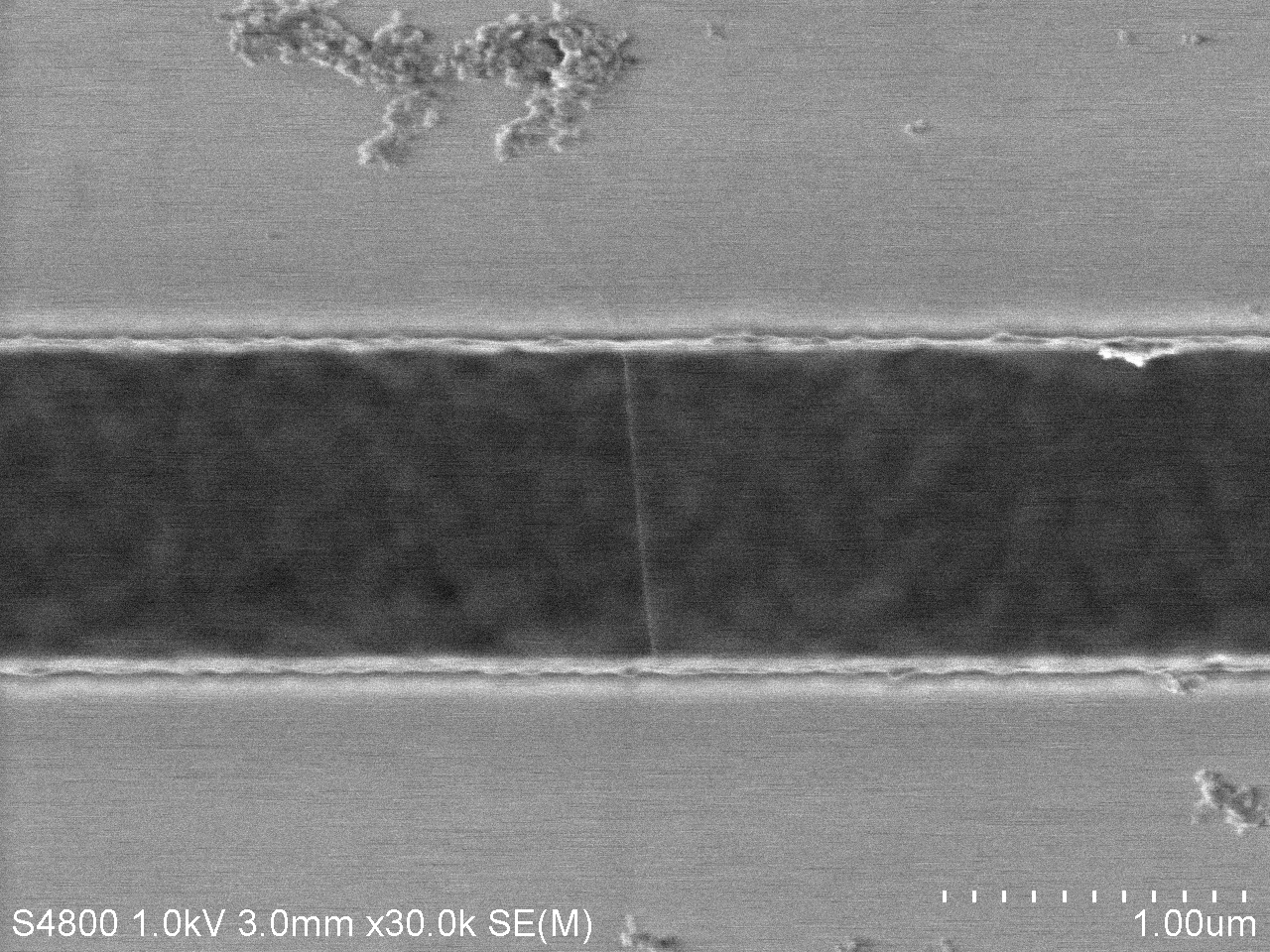

Supplement: Supplementary file 3 — Source Data [file 41467_2022_30508_MOESM3_ESM.zip › Source Data/Figure 1/1b/functionalized swnt 1_q007.tif]

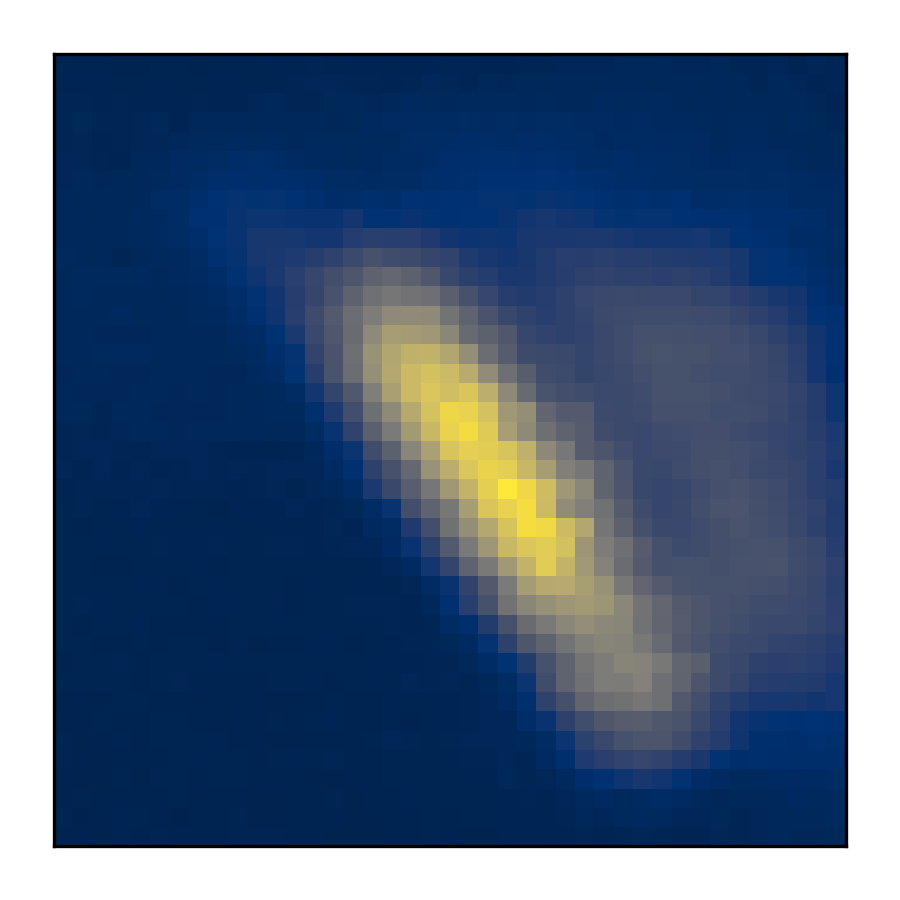

Supplement: Supplementary file 3 — Source Data [file 41467_2022_30508_MOESM3_ESM.zip › Source Data/Figure 1/1d/map_0.png]

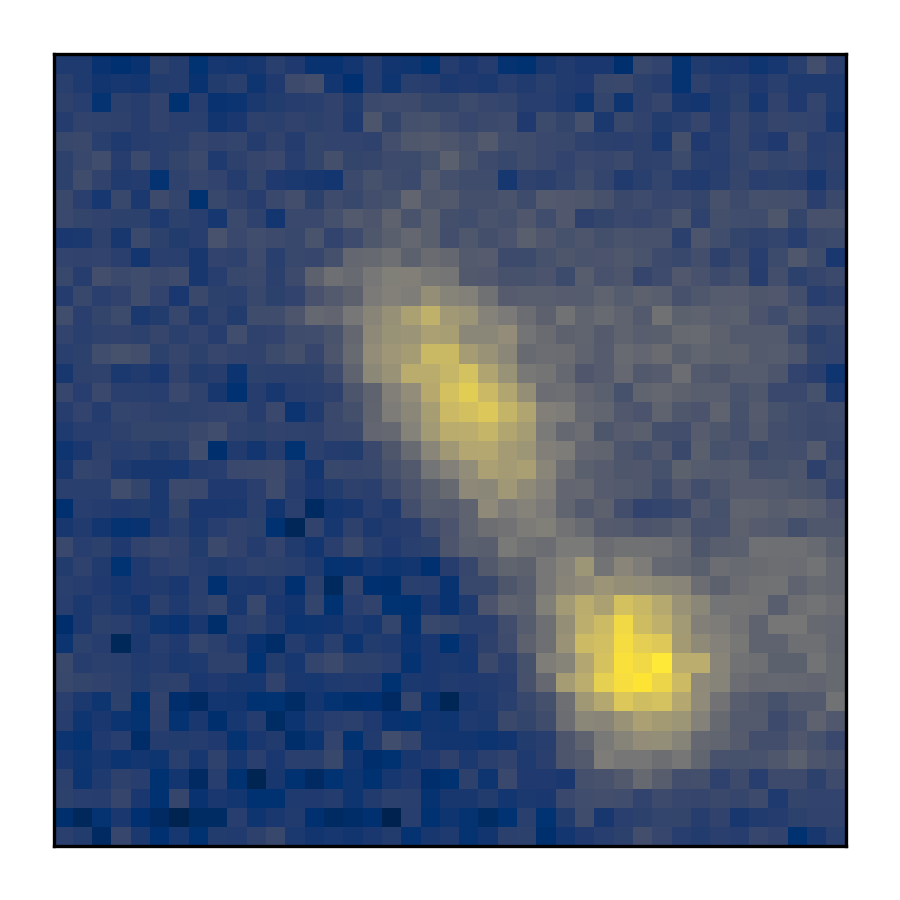

Supplement: Supplementary file 3 — Source Data [file 41467_2022_30508_MOESM3_ESM.zip › Source Data/Figure 1/1e/map_1.png]

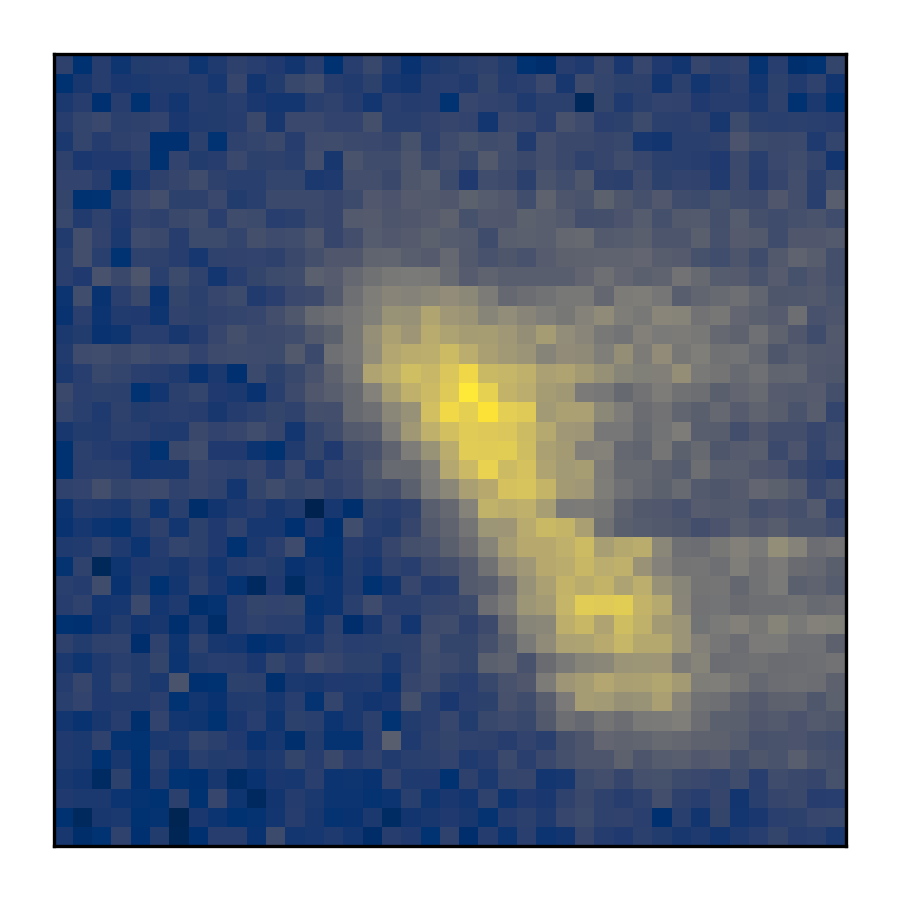

Supplement: Supplementary file 3 — Source Data [file 41467_2022_30508_MOESM3_ESM.zip › Source Data/Figure 1/1f/map_2.png]

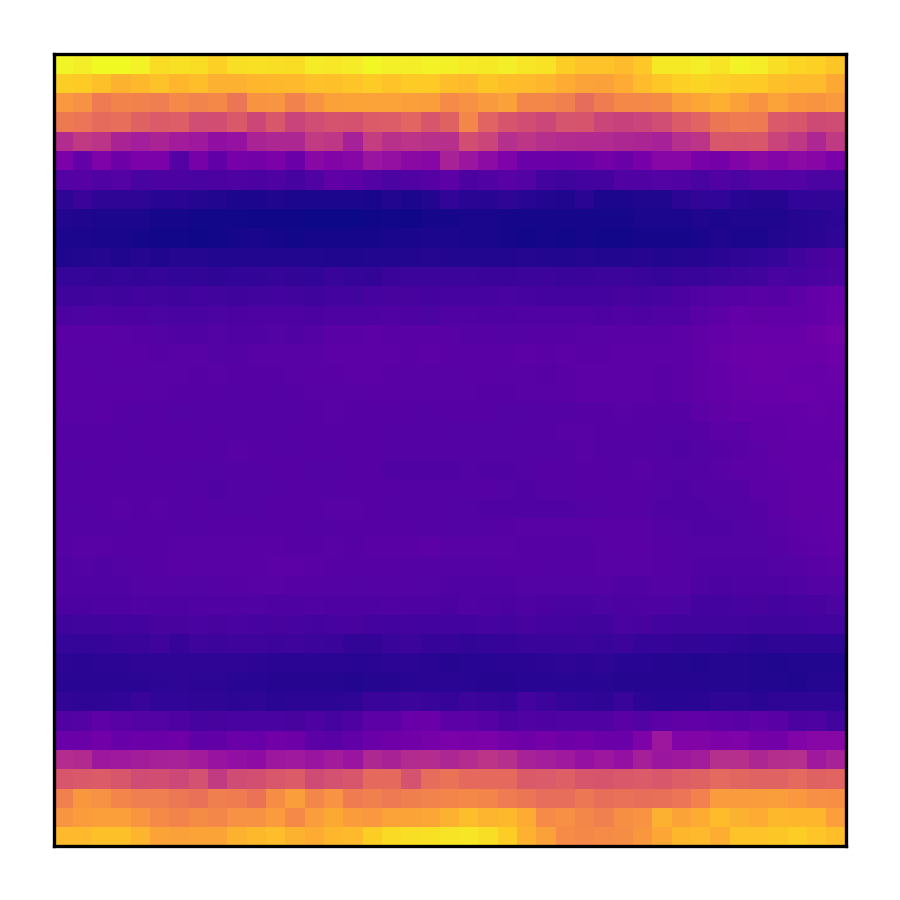

Supplement: Supplementary file 3 — Source Data [file 41467_2022_30508_MOESM3_ESM.zip › Source Data/Figure 1/1g/190519XY02 Chip#8-780nm-CNT#24_01 CW_780nm_100uW_90deg_500ms.png]

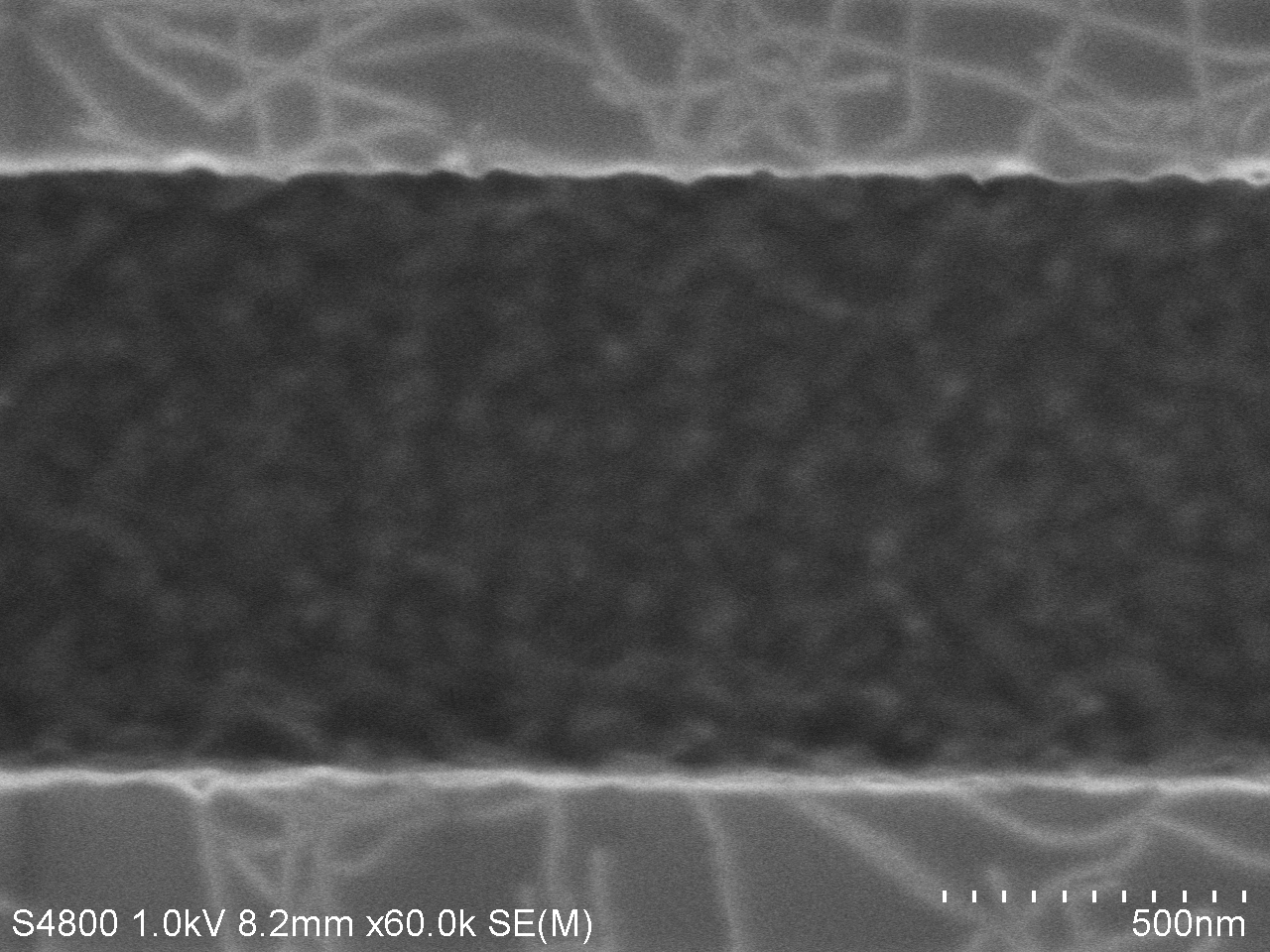

Supplement: Supplementary file 3 — Source Data [file 41467_2022_30508_MOESM3_ESM.zip › Source Data/Figure S1/TC5KS77_1000nm_1st_dip_q011.tif]

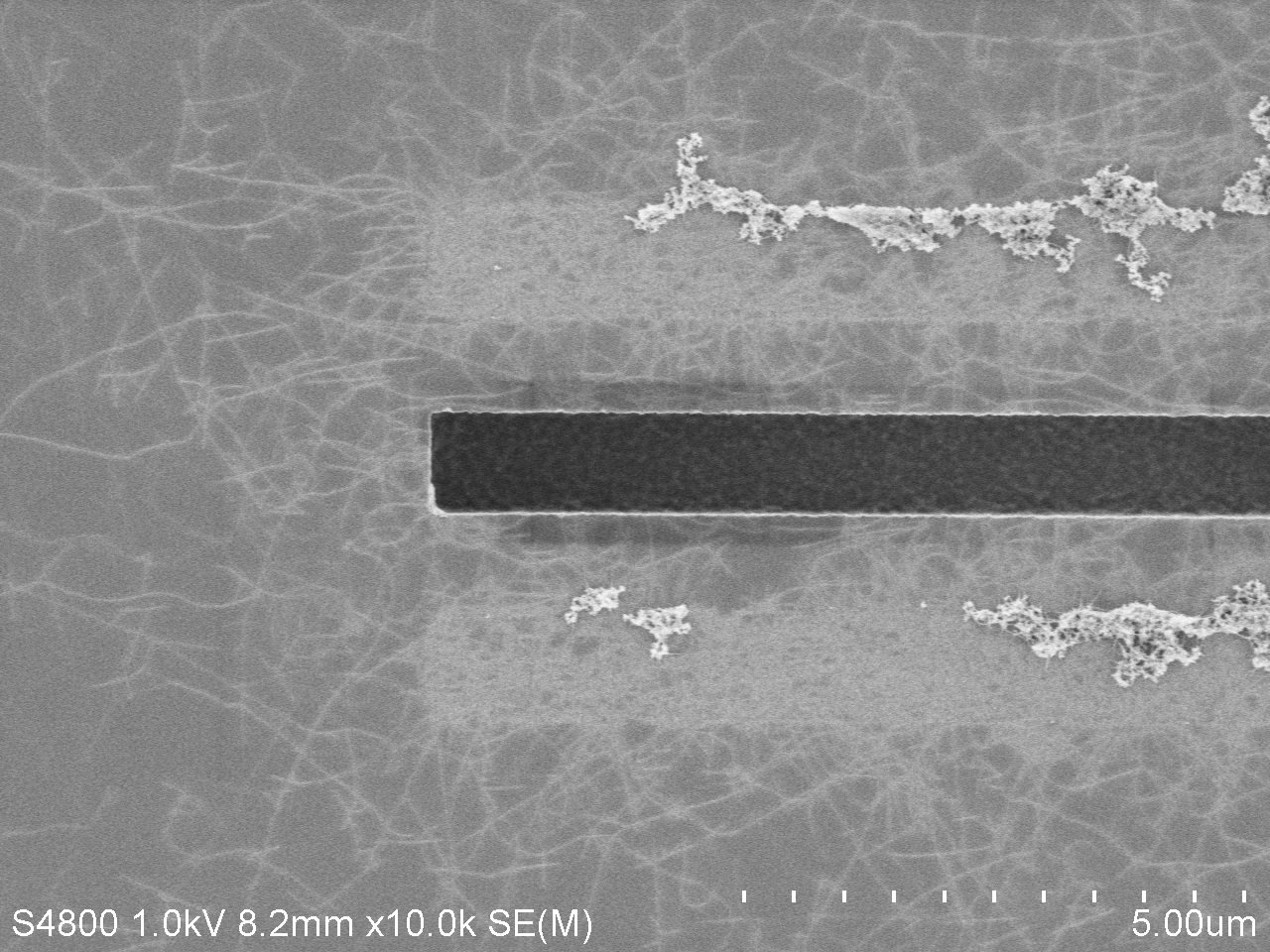

Supplement: Supplementary file 3 — Source Data [file 41467_2022_30508_MOESM3_ESM.zip › Source Data/Figure S1/TC5KS77_1000nm_1st_dip_zoomedout_q012.tif]

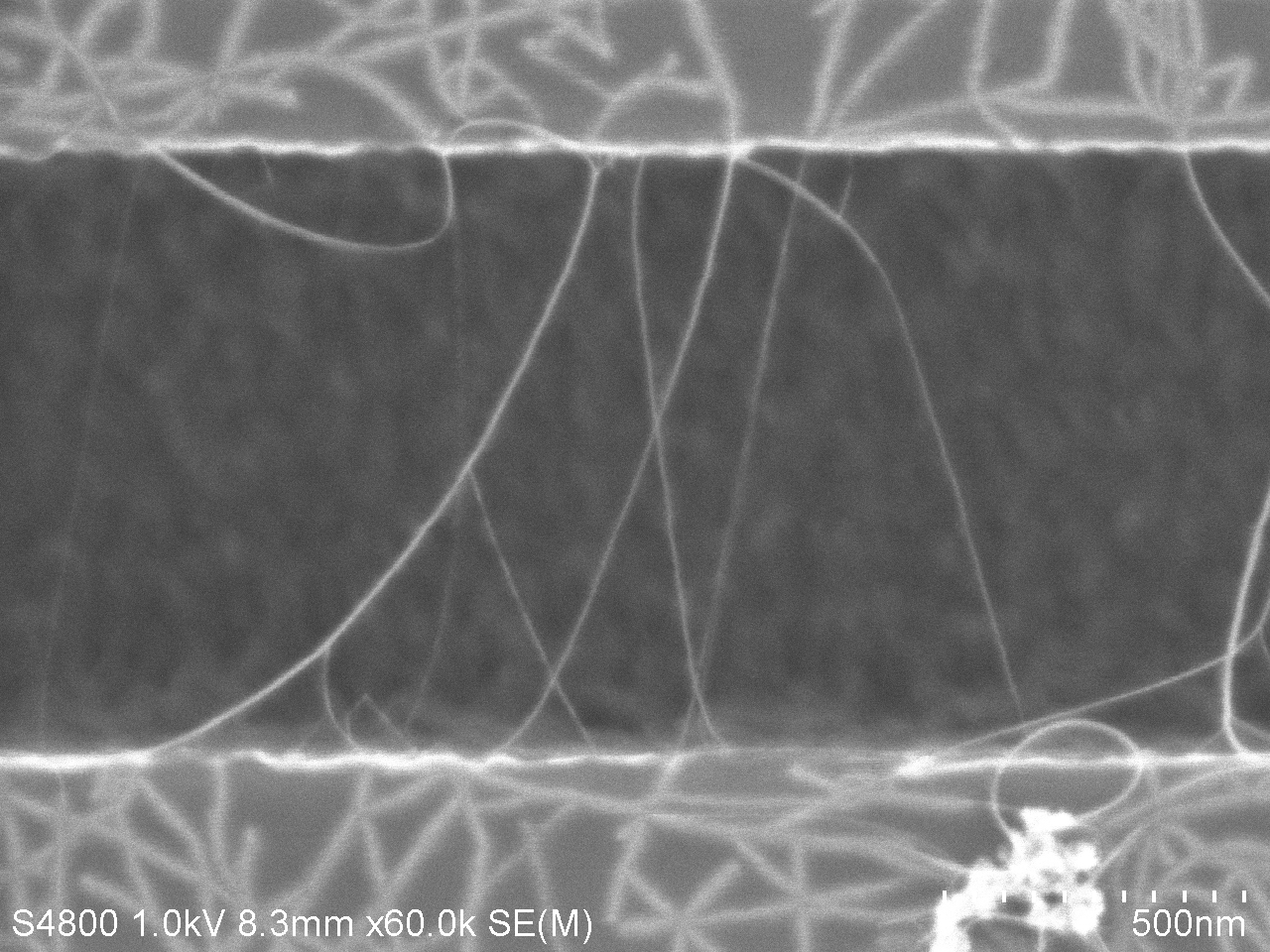

Supplement: Supplementary file 3 — Source Data [file 41467_2022_30508_MOESM3_ESM.zip › Source Data/Figure S1/TC5KS77_1000nm_1st_q002.tif]

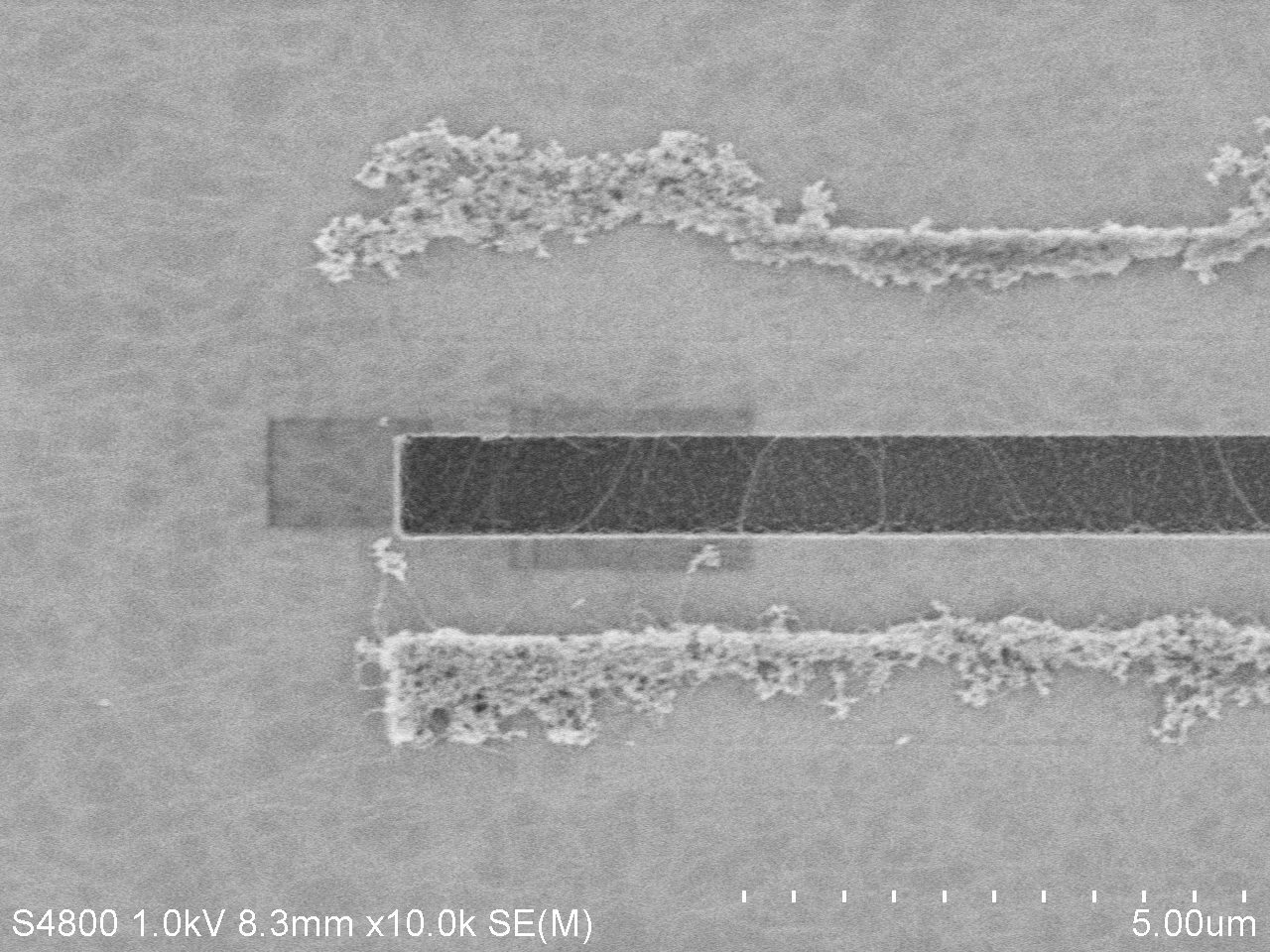

Supplement: Supplementary file 3 — Source Data [file 41467_2022_30508_MOESM3_ESM.zip › Source Data/Figure S1/TC5KS77_1000nm_1st_zoomout_q003.tif]

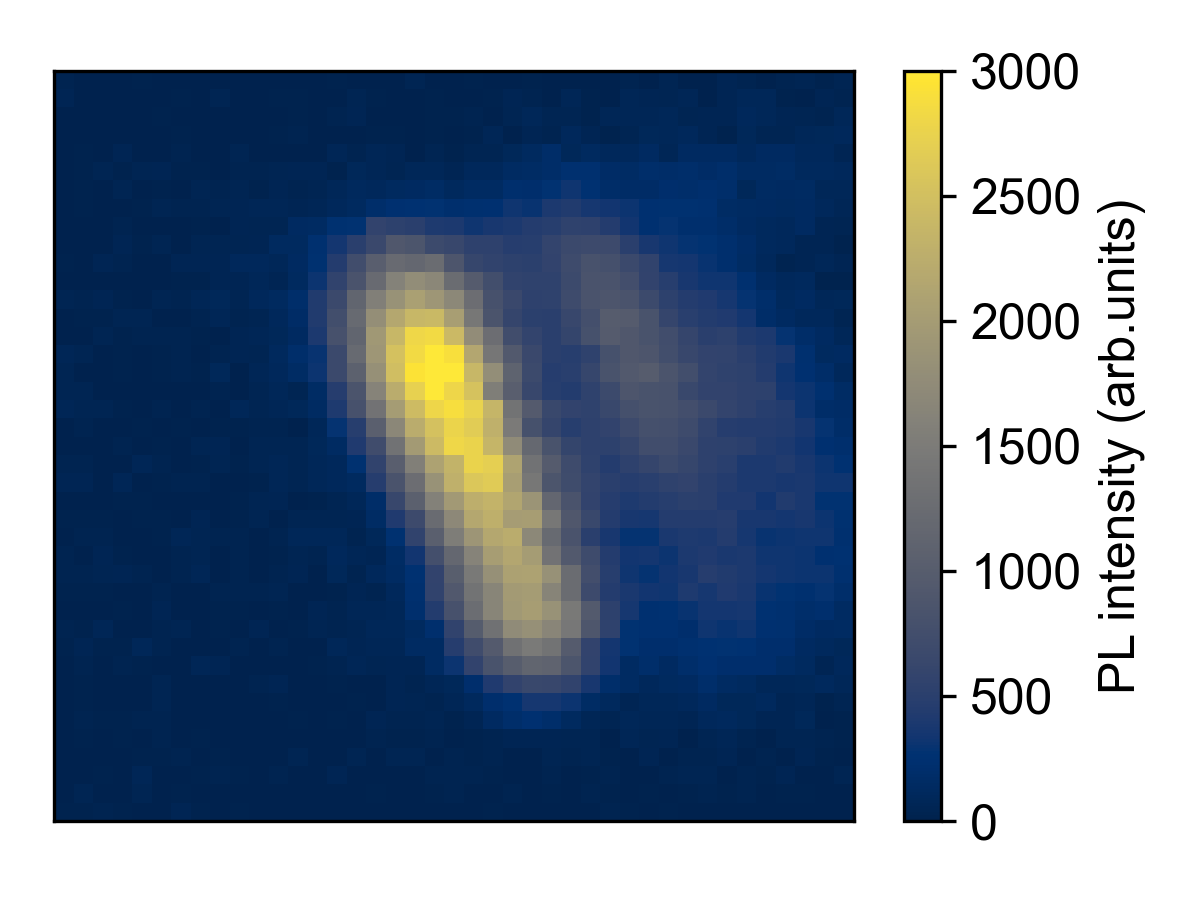

Supplement: Supplementary file 3 — Source Data [file 41467_2022_30508_MOESM3_ESM.zip › Source Data/Figure S15/a/PL_fullXYSP02_at1199nm Z.png]
